# Advancing Ultrasonic Defect Detection in High-Speed Wheels via UT-YOLO

**DOI:** 10.3390/s24051555

**Published:** 2024-02-28

**Authors:** Qian Zhang, Jianping Peng, Kang Tian, Ai Wang, Jinlong Li, Xiaorong Gao

**Affiliations:** 1 School of Physical Science and Technology, Southwest Jiaotong University, Chengdu 610036, China; zhangqian94@my.swjtu.edu.cn (Q.Z.); tiankang@my.swjtu.edu.cn (K.T.); jinlong_lee@swjtu.edu.cn (J.L.); gxrr@vip.163.com (X.G.); 2 Chengdu Lead Science & Technology Co., Ltd., Chengdu 610073, China; ai.wang.ms@gmail.com

**Keywords:** ultrasonic testing, B-scan images, defect detection, UT-YOLO

## Abstract

In the context of defect detection in high-speed railway train wheels, particularly in ultrasonic-testing B-scan images characterized by their small size and complexity, the need for a robust solution is paramount. The proposed algorithm, UT-YOLO, was meticulously designed to address the specific challenges presented by these images. UT-YOLO enhances its learning capacity, accuracy in detecting small targets, and overall processing speed by adopting optimized convolutional layers, a special layer design, and an attention mechanism. This algorithm exhibits superior performance on high-speed railway wheel UT datasets, indicating its potential. Crucially, UT-YOLO meets real-time processing requirements, positioning it as a practical solution for the dynamic and high-speed environment of railway inspections. In experimental evaluations, UT-YOLO exhibited good performance in best recall, mAP@0.5 and mAP@0.5:0.95 increased by 37%, 36%, and 43%, respectively; and its speed also met the needs of real-time performance. Moreover, an ultrasonic defect detection data set based on real wheels was created, and this research has been applied in actual scenarios and has helped to greatly improve manual detection efficiency.

## 1. Introduction

Wheels play a pivotal role as integral components of trains, directly influencing the safety and reliability of train operations [1]. With the rapid development and increasing speed of high-speed trains, corresponding safety risks are also escalating. The quality and structural condition of high-speed rail wheels have a direct impact on the safety and overall performance of an entire train. Operating at high speeds exposes the wheels to various external factors, including wear, impacts, and fatigue cracks, which will lead to deformation, damage, or even detachment, particularly under high-load and -frequency conditions. Wheel defects can lead to severe traffic accidents.

Non-destructive testing is a method that preserves the integrity of the object being examined [2], primarily being used for detecting defects in materials, components, and equipment. Traditional non-destructive testing (NDT) methods encompass ultrasonic testing (UT) [3], magnetic particle testing (MT) [4], and penetrant testing (PT) [5]. In the railway industry, owing to the unique environment, nearly all inspections rely on non-destructive testing. X-ray computed tomography has been employed to address the reconstruction of rolling contact fatigue cracks in rails [6], and various ultrasonic testing methods have been applied in axle defect detection [7,8]. Given the complex structures of wheels and the multitude of components, there are a variety of detection methods [9,10,11].

Traditional wheel defect detection methods predominantly hinge on visual inspection and ultrasonic testing. Visual inspection, however, is inefficient and prone to inaccuracies. Ultrasonic testing, relying on the principles of ultrasonic wave propagation in materials, involves emitting ultrasonic waves and capturing reflected signals. This approach enables the detection of various defects within wheels, such as cracks, inclusions, and porosities. Characterized by high sensitivity and resolution, this technology proves effective in identifying minute defects, offering early warnings of potential issues and contributing to the prevention of safety hazards arising from wheel failures.

The application of ultrasonic testing to train wheels goes beyond routine periodic inspections; it is also applicable in emergencies, such as post-collision or other external impact scenarios. Continuous monitoring of wheel health empowers operators to implement timely maintenance and repair measures, ensuring the safety and stability of trains during high-speed operations. However, due to the huge quantity of data, relying solely on manual detection is too inefficient. Traditional detection methods rely more on fixed methods. Matching and efficiency are difficult to guarantee. At the same time, subject to the limitations of expert knowledge, the detection process is relatively complicated. Therefore, a set of novel methods that can meet the above needs continues to be applied and promoted.

Deep learning technology represents a significant advancement in enhancing the efficiency and accuracy of computer vision as well as NDT. Its application extends to the automated analysis of various NDT data, including ultrasonic signals, magnetic particle images, and penetrant images, thereby substantially improving defect detection accuracy. Specifically, in ultrasonic wheel defect detection, deep learning technology offers several advantages. Firstly, it facilitates the automatic analysis of ultrasonic signals, thereby significantly enhancing detection efficiency. Secondly, it can extract intricate features from ultrasonic signals that may be challenging for human recognition, leading to a substantial improvement in detection accuracy. Additionally, deep learning technology contributes to cost reduction and minimizes manual intervention in the testing process. One notable strength of deep learning models is their ability to classify defects into different types, accurately locate defect positions, and measure defect sizes. These affordances not only streamline the analysis process but also provide a comprehensive understanding of the detected defects. By annotating ultrasound B-scan images, we can annotate a defect, which will give us a UT dataset. By learning from a large quantity of effective data, a model can efficiently detect data.

Despite these advantages, the application of deep learning technology in ultrasonic wheel defect detection still encounters certain challenges. One of the primary challenges is the creation of high-quality datasets. The performance of deep learning models heavily relies on the quality and diversity of the training data. Ensuring that the datasets used for training are representative of real-world scenarios is essential for achieving robust and reliable defect detection. Another challenge lies in the high complexity of deep learning models. While these models exhibit remarkable capabilities, their complexity can lead to challenges in interpretability and the need for significant computational resources. Striking a balance between model complexity and interpretability is crucial for practical implementation and widespread adoption. While deep learning technology has immense potential for revolutionizing ultrasonic wheel defect detection by significantly improving efficiency and accuracy, addressing challenges related to dataset creation and model complexity is essential for realizing its full benefits in real-world applications. To this end, we need to explain the categories of defects. In this work, the defect data are divided into four different forms according to the depth of scanning, namely, surface, near-surface, internal, and wheel plate cracks.

Given that natural images seldom exhibit exaggerated aspect ratios, standard deep-learning approaches may fall short when applied directly to ultrasonic images. Hence, this article explores specialized preprocessing techniques aimed at enhancing the compatibility between deep learning models and the intricate features inherent in ultrasonic wheel defect B-scan images. By addressing these specific challenges through dedicated preprocessing methods, the goal is to optimize the learning capacity of deep neural networks, fostering improved accuracy in detecting defects. This approach is crucial in bridging the gap between conventional image-processing methodologies and the distinctive characteristics of ultrasonic datasets, ultimately enhancing the efficacy of defect detection in high-speed railway train wheels. The design and training of deep learning models need to be tailored to accommodate these distinctive characteristics, ensuring optimal performance in detecting ultrasound defects. The main contributions of this paper are as follows:

- An ultrasonic defect detection data set based on real wheels was created, and the defects were subdivided according to different categories;
- An advanced UT-YOLO network was proposed to increase a network’s ability to perceive small defects and detection;
- This research has been applied in actual scenarios and has helped to greatly improve manual detection efficiency.

## 2. Related Work

### 2.1. Traditional Defect Detection

Traditional defect detection methods are based on the characteristics of an object, such as color, size, shape, etc., and use image-processing methods and traditional machine learning methods for detection. Among them, the representative target detection methods include Scale-Invariant Feature Transform (SIFT) [12], Histogram of Oriented Gradients (HOG) [13], Oriented FAST and Rotated BRIEF (ORB) [14], and Harr [15]. It is necessary to use a pre-designed template to slide the window on the target image, and the steps are cumbersome. The extracted object features are all low-level, manually selected features, so they are not conducive to accurate detection and offer poor real-time detection. Moreover, these methods are mostly used for obvious features in natural images which have rich information. For these simple tasks, generally, Prewitt, Sobel, and Canny can be used to find solutions. However, in practice, especially in complicated wheel defect detection situations, noise is very serious, and much depends on the accuracy of the equipment and the skilled operators. All in all, due to the limitations of many factors, ultrasonic detection images are often not used in this type of detection method.

While conventional methods exhibit proficiency in straightforward industrial scenarios governed by clear rules, they encounter challenges when confronted with the intricacies and diversities inherent in real-world defects. Preceding the era of deep learning, object detection heavily relied on meticulously crafted features. Although this approach attained some degree of success, it grappled with notable limitations. For instance, the manual design of features often necessitated subjective decisions, resulting in inconsistencies and a lack of generalizability. The manual approach struggled to accommodate the vast diversity and complexity of real-world objects, and as the number of object categories and image variations expanded, the manual design process became increasingly cumbersome and impractical. Moreover, the broad spectrum of potential defects demanded multiple sets of pre-defined templates, constraining these methods’ capacity to identify novel and unexpected issues. Consequently, when confronted with intricate data, traditional methods frequently falter, necessitating laborious post-processing efforts.

### 2.2. Deep-Learning-Based Defect Detection

The rise of deep learning has transformed object detection, offering notable advantages over traditional methods. Convolutional neural networks (CNNs) excel at extracting high-level features from images, overcoming the limitations of manual feature crafting. Their seamless integration of feature extraction, selection, and classification enhances overall efficiency and effectiveness. Scholarly work consistently demonstrates the prowess of deep learning in defect detection, particularly in industrial settings.

The R-CNN network was proposed by Girshick et al. [16] in 2013. It is a landmark effort in the field of object detection. He et al. proposed SPP-Net [17], which no longer extracts features from all candidate areas and only extracts features of an entire image once, which greatly reduces the amount of calculation. Faster-RCNN [18] was the most representative model in the R-CNN series that was proposed by Ren et al. Because the Region Proposal Network (RPN) realized end-to-end training and testing of a network, detection accuracy was highly improved. However, Faster-RCNN is generally more complex, and its performance on small objects is not very impressive. He et al. proposed Mask RCNN [19], which has high accuracy in detection, but at the same time, instance segmentation will increase label price. The biggest problem for practical use is that the speed of the algorithm still cannot meet the industrial real-time detection requirements. In the industrial field, some researchers have adjusted the common detection frameworks to suit the detection of industrial data. Liu et al. [20] improved Faster-RCNN by utilizing cascade heads to detect metal defects. Cha et al. [21] changed the backbone to improve the detection time for bridge defects based on Faster-RCNN. He et al. [22] improved detection accuracy using multi-scale feature diffusion. The pursuit of greater detection speed has favored a large number of one-stage algorithms.

Redmon et al. [23] proposed the first one-stage detector, YOLO v1, which uses a multi-scale region centered on a grid to replace the RPN, greatly improving detection speed and meeting the needs of real-time detection but not in terms of accuracy. A novel object detection method called SSD [24] was proposed in 2016, combining the advantages of both Faster-RCNN and YOLO v1. It ensures high-precision detection while taking into account detection speed by using multi-scale regional features for regression, particularly via combining high-level and low-level feature maps. YOLO v2 [25] employs Darknet-19 as a backbone network of a model to extract features and reduce the computational complexity of the model. In addition, it further improves detection speed and accuracy by adding batch normalization, multi-scale training, and K-means dimension clustering after each convolutional layer. YOLO v3 [26] introduced several key advancements over its predecessors, leading to improved performance in both speed and accuracy. One of its most significant contributions is the Darknet-50 residual network, a newly designed architecture that utilizes residual connections for deeper feature extraction. This network was combined with the feature pyramid network (FPN) [27], which enabled multi-scale fusion prediction and detection on three different-scale feature maps. YOLO v4 [28] uses CSPDarkNet53 as a backbone network and optimizes to varying degrees using data processing, training methods, activation functions, loss functions, etc., to achieve the best detection effect at a given time. Li et al. [29] provided an end-to-end solution for the surface defect detection of steel strips based on improved YOLO. Zhang et al. [30] improved YOLO v3 by introducing a novel transfer learning method with fully pre-trained weights from a geometrically similar dataset to detect bridge surface damage.

However, all algorithms are based on the premise of sufficient data. However, in actual situations, especially in industrial environments, due to the particularity of application scenarios, sufficient and balanced labeled data sets are difficult to obtain, even if ordinary data augmentation cannot completely solve this problem. A novel automated defect synthesis network called Defect-GAN was designed by Zhang et al. [31] to generate realistic and diverse defect samples for training accurate and robust defect inspection networks. Valente et al. [32] used synthetic training data by simulating two types of print effects with image-processing and computer graphic techniques.

Building upon YOLO v5s as the baseline, this work introduces UT-YOLO, a novel ultrasonic testing defect detection method. UT-YOLO incorporates several key advancements over the baseline model, namely, enhanced backbone architectures with Swin-Transformer [33] and ResNet [34] variants, improved neck modules like BiFPN [35] for better feature aggregation, a dedicated multi-detection head for the accurate localization of small defects, a novel attention module for enhanced feature representation, and a practical pre-processing pipeline specifically tailored for real-world ultrasonic B-scan images, ensuring robust and effective defect detection in industrial applications.

## 3. Methodology

**Figure 1:**
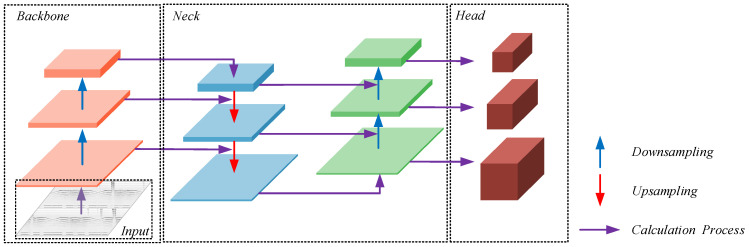
The architecture of YOLO v5.

The basic architecture of YOLO v5 is shown in Figure 1. The whole operation process is as follows: Firstly, the images are readied for feature extracted by the backbone network, named CSPDarkNet53 [28]; then, the features in the neck section are separated into different sizes for feature fusion to obtain more feature information. After that, the different detection heads are designed, which allows the algorithm to fulfill the specific requirements of object detection.

### 3.1. Backbone

**Figure 2:**
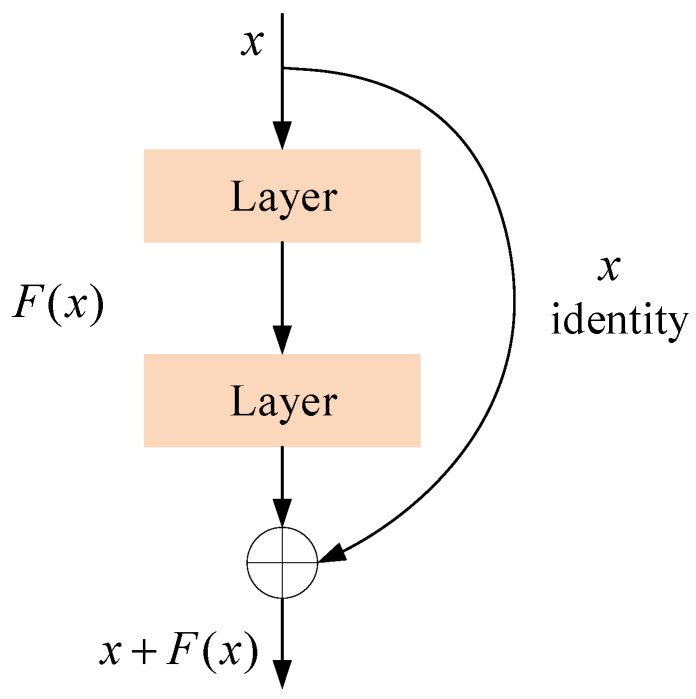
The structure of a residual connection.

The architecture of the backbone is designed for feature extraction, but its efficiency should be given more attention. Due to this consideration, some novel backbone structures were applied in this task. The defects from ultrasonic testing B-scan images appear in different regions, so a useful feature extraction backbone should be designed to solve this problem. Regarding the wheels of railway trains, the defects are always small and noise-like, making them hard to identify easily. Figure 2 shows a typical residual connection, which is widely used in the design of backbone improvements. By introducing residual connections, the neural network is allowed to skip certain levels of learning, thereby alleviating the vanishing and exploding gradient problems and making training easier. This network also supports deeper networks. Traditional deep neural networks suffer from the problem wherein the training difficulty increases as the depth of the network increases. The residual structure allows building deeper networks by adding more layers without degradation issues.

**Figure 3:**
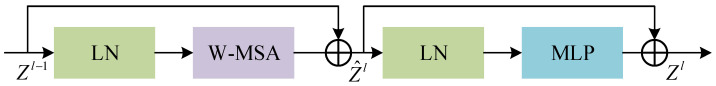
The structure of Swin-Transformer.

Figure 3 shows the structure of the Swin-Transformer block. It allows a model to better capture global relationships among pixels in an image and avoids full connection to reduce calculation costs, affordances that are of vital importance in object detection, especially in ultrasonic defect testing. At the same time, by stacking multiple transformer blocks, the network is able to learn more complex and abstract feature representations.

Here, $Z^{l-1}$ represents the output of layer l−1 or the input of layer l. The information passes from one layer to the next layer sequentially.

Firstly, the input is linearly transformed and normalized; then, it is passed through Weighted Multi-Head Self Attention (W-MSA), which focuses on a different area of the input rather than the global average. Secondly, the output will be added by the residual connection. The new output will repeat another Layer Normalization (LN) step and then connect with a Multi-Layer Perceptron (MLP) to fit the complicated input data. Finally, the residual connection will be used again to improve model stability. It is usually used as a repeated block, which helps alleviate vanishing or exploding gradient problems during training, making the network easier to train and optimize. In wheel defect feature extraction, after convolution and residual connection, the feature extraction module of Swin-Transformer can be superimposed multiple times to obtain more semantic information, which plays an important role in distinguishing background noise and defects.

### 3.2. Neck

**Figure 4:**
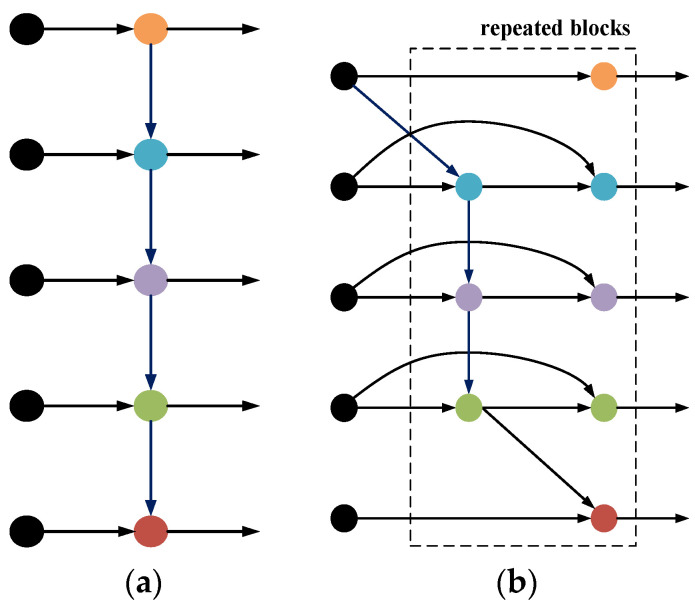
(**a**) Schematic diagram of PANet; (**b**) schematic diagram of BiFPN.

BiFPN, a bidirectional feature propagation network, facilitates the bidirectional flow of information, allowing features to traverse from high-level layers to low-level layers, as well as enabling the propagation of low-level features to high-level layers. This dual-directional information exchange enhances the model’s ability to capture semantic information across various feature levels, thereby augmenting the model’s perceptual capabilities towards the target. In contrast, PANet adopts unidirectional propagation, limiting the flow of information to a single direction. The corresponding structure is shown in Figure 4.

**Figure 5:**
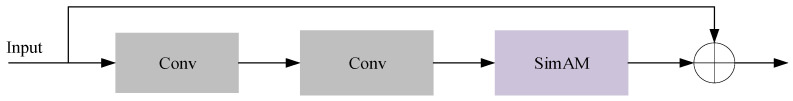
The diagram of SimAM.

Another noteworthy aspect is that BiFPN was meticulously designed to mitigate computational complexity without compromising model efficacy. It achieves this by judiciously fusing features at distinct levels, thereby sidestepping the computational overhead associated with full connections. In contrast, PANet exhibits a more intricate design that could entail a higher computational burden. Nevertheless, a novel enhancement of BiFPN made by incorporating the Simple, Parameter-Free Attention Module (SimAM) [36] was proposed. This addition enables the network to selectively concentrate on varying levels of features, thus refining its capacity for nuanced feature extraction. A diagram of SimAM is shown in Figure 5.

### 3.3. Head

**Figure 6:**
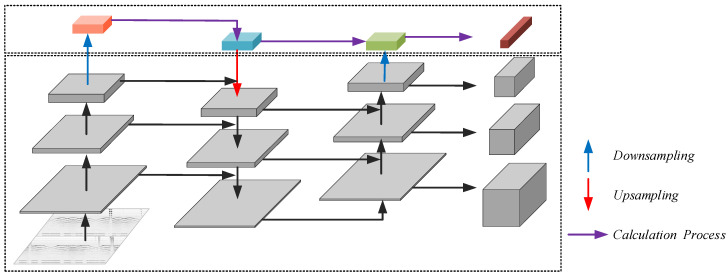
The new small-object detection head.

Given that ultrasonic testing typically involves small targets, the existing detection heads may struggle to meet the required standards. Consequently, this study enhances the number of detection heads and simultaneously augments the depth of the detection heads within the target detection model. The objective is to enhance the model’s discernment of intricate scenes and nuanced features in pursuit of more effective performance. The new structure is shown in Figure 6.

The added detection head is shown in Figure 6. The structure in the dotted box in the lower half is consistent with that in Figure 1 but is rendered in gray. On this basis, in the upper block diagram of the network, a small target detection layer was added. Using color marking, the structure is used for the detection of small targets.

### 3.4. Loss Function

The original YOLOv5 loss function generally consists of three components, as represented by Equation (1). The first term quantifies localization loss, evaluating the accuracy of the model’s predictions for the position of the target bounding box. The second term corresponds to confidence loss, measuring how accurately the model predicts the presence of a target. The final term assesses the accuracy of the classification

(1) $$L_{\mathrm{ori}}\left(t_{p},t_{\mathrm{gt}}\right)=\sum_{k=0}^{K}\left[\alpha_{k}^{w}\alpha_{\mathrm{box}}\sum_{i=0}^{S^{2}}\sum_{j=0}^{B}I_{k_{ij}}^{obj}L_{\mathrm{CIoU}}+\alpha_{\mathrm{obj}}\sum_{i=0}^{S^{2}}\sum_{j=0}^{B}I_{k_{ij}}^{obj}L_{\mathrm{obj}}+\alpha_{\mathrm{cls}}\sum_{i=0}^{S^{2}}\sum_{j=0}^{B}I_{k_{ij}}^{obj}L_{\mathrm{cls}}\right]$$

where K, $S^{2}$, and B are the number of output features, the number of grids, and the number of anchors, respectively. $\alpha_{*}$ is the weight for different sections; $\alpha_{\mathrm{box}}$, $\alpha_{\mathrm{obj}}$, and $\alpha_{\mathrm{cls}}$ are set as 0.05, 0.3, and 0.7, respectively. $I_{k_{ij}}^{obj}$ represents whether the i-th cell, the j-th anchor box in the k-th feature map, is a positive sample. If it is positive, it is defined as 1; otherwise, it is set as 0. $t_{p}$ and $t_{\mathrm{gt}}$ are the prediction vector and ground truth (GT), and $\alpha_{k}^{w}$ is the weight for different sizes of the output.

Equation (2) is the new loss function called $L_{\mathrm{UT}}$, which is designed for UT defect detection. The localization loss is replaced by $L_{\mathrm{EIoU}}$.

(2) $$L_{\mathrm{UT}}\left(t_{p},t_{\mathrm{gt}}\right)=\sum_{k=0}^{K}\left[\alpha_{k}^{w}\alpha_{\mathrm{box}}\sum_{i=0}^{S^{2}}\sum_{j=0}^{B}I_{k_{ij}}^{obj}L_{\mathrm{EIoU}}+\alpha_{\mathrm{obj}}\sum_{i=0}^{S^{2}}\sum_{j=0}^{B}I_{k_{ij}}^{obj}L_{\mathrm{obj}}+\alpha_{\mathrm{cls}}\sum_{i=0}^{S^{2}}\sum_{j=0}^{B}I_{k_{ij}}^{obj}L_{\mathrm{cls}}\right]$$

**Figure 7:**
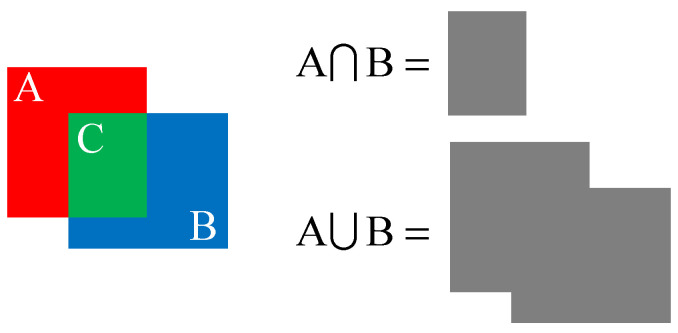
The schematic diagram of IoU.

Intersection over Union (IoU) is used to evaluate the quality of object detection models. A schematic diagram of IoU is shown in Figure 7.

Here, the value of IoU should be described as in Equation (3).

(3) $$\mathrm{IoU}=\frac{A\cap B}{A\cup B}$$

**Figure 8:**
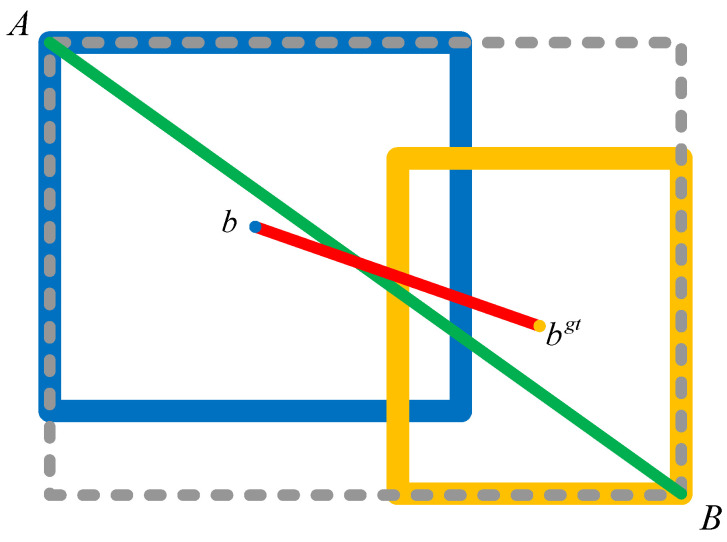
A schematic diagram of prediction box (blue) and GT (yellow).

Simply put, the overlapping region of A and B is C, which is denoted as A∩B. The area of A+B−C is denoted as A∪B. The ratio of A∩B and A∪B is IoU, which is an indicator of prediction accuracy. In the original loss function, a complete IoU (CIoU) was used to solve the special cases with the same central point but different ratios of height to width. Equations (4)–(6) define the loss of CIoU

(4) $$L_{\mathrm{CIoU}}=1-\mathrm{CIoU}$$

(5) $$\mathrm{CIoU}=\mathrm{IoU}-\left(\frac{\rho^{2}\left(b,b^{gt}\right)}{c^{2}}+\alpha \nu \right)$$

(6) $$\nu =\frac{4}{\pi^{2}}{\left(arctan\frac{w^{gt}}{h^{gt}}-arctan\frac{w}{h}\right)}^{2}$$

where α is weight and the ν is the parameter for measuring the ratio of height to width. A schematic diagram is shown in Figure 8: the green line (AB) is the parameter c, which signifies the diagonal distance of the minimum bounding box (the gray, dotted one) capable of encompassing both the predicted box and the GT box. Meanwhile, the parameter ρ is the Euclidean distance between two center points b and $b^{gt}$, which are indicated by the red line.

At the same time, the definition of ν is shown in Equation (7):

(7) $$\nu =\frac{4}{\pi^{2}}{\left(arctan\frac{w^{gt}}{h^{gt}}-arctan\frac{w}{h}\right)}^{2}$$

In Equation (2), the penalty term of EIoU consists of separating the influence factors of the aspect ratio based on the penalty term of CIoU to calculate the length and width of the target frame and anchor frame, respectively. So, $L_{\mathrm{EIoU}}$ is changed according to Equation (8).

(8) $$L_{\mathrm{EIoU}}=1-\mathrm{IoU}+\frac{\rho^{2}\left(b,b^{gt}\right)}{c^{2}}+\frac{\rho^{2}\left(w,w^{gt}\right)}{c_{w}^{2}}+\frac{\rho^{2}\left(h,h^{gt}\right)}{c_{h}^{2}}$$

Therefore, $L_{\mathrm{EIoU}}$ can provide a more accurate assessment when an object’s position is imprecise or slightly offset. However, to use $L_{\mathrm{EIoU}}$ as the loss function, the dataset should be more closely scrutinized, especially regarding the balance between various types of data.

To strike a balance between real-time requirements and computational complexity, the original YOLOv5 algorithm was chosen as the baseline model. This work enhanced the baseline model by (1) utilizing dataset-preprocessing methods that incorporate the characteristics of ultrasonic B-scan data, enhancing the model’s compatibility with neural networks; (2) employing a combination of ResNet, Swin-Transformer, and BiFPN structures for feature extraction to capture richer feature information, thereby increasing the capability of feature extraction; (3) introducing SimAM and a dedicated small-object detection head to further optimize the detection accuracy for small objects; and (4) enhancing the accuracy of bounding box regression through the refinement of the loss function’s design.

## 4. Experiments and Results

Diverging from natural images, ultrasonic detection images primarily convey intensity information, rendering them particularly susceptible to noise interference. Moreover, as the focus of this study is on detecting defects in wheels, the acquired data hold exceptional value. The experiments were conducted alongside an exploration of datasets and various experiments.

### 4.1. Dataset

**Figure 9:**
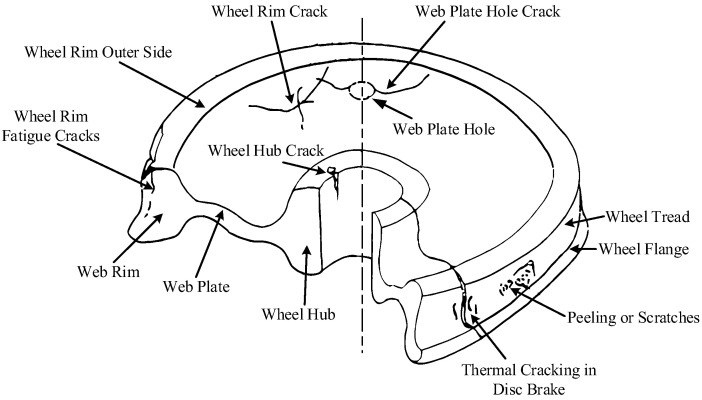
Diagram of typical wheel defects.

This work was evaluated with reference to a real dataset, which was collected with phased array UT (PAUT) and transmit–receive (TR) probes to attain B-scan images about four different defects according to different depths, surfaces near other surfaces, and internal and rim cracks in different service depots using the LU system [37]. The entire dataset of UT defects consisted of around 15,000 B-scan images, in which peeling, scratches, and cracks in different positions and depths were recorded. It is hard to find different real images with which to show the different types of defects, especially some inner defects, so a diagram of typical wheel defects is shown in Figure 9.

**Figure 10:**
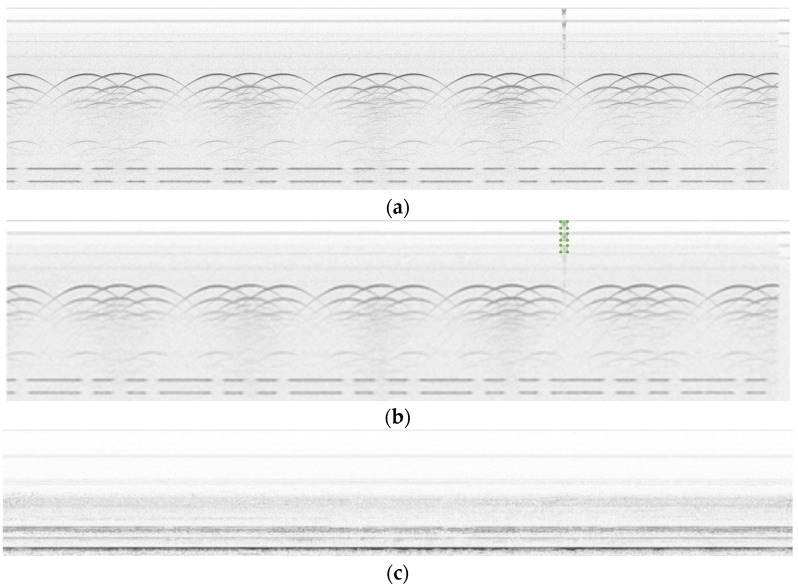
Examples of (**a**) original UT B-scan with a defect; (**b**) original UT B-scan with a defect and annotation; and (**c**) original UT B-scan data without defects.

UT is a very useful solution for detecting inner defects, but analyzing surface and sub-surface areas is not its strength because of the large amount of initial wave interference at the contact surface, as well as the noise; last but not the least, due to the particularity of the wheel structure, the collection is generally completed in one cycle, so the aspect ratio of the collected images will be very large. Examples of the original collection data are shown in Figure 10. Image (a) below depicts a surface defect, and the periodic signal represents the wheel plate hole; there are six holes in a wheel.

**Figure 11:**
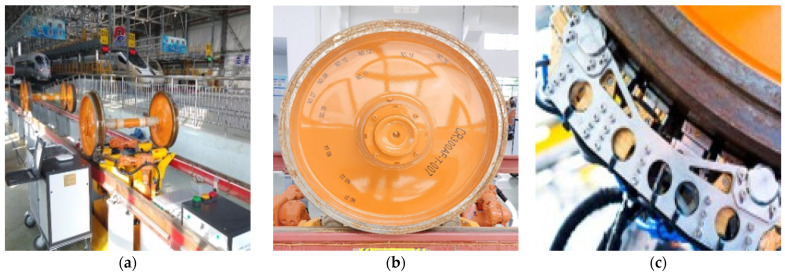
Typical scenarios: (**a**) wheel detection in service depots; (**b**) wheel; (**c**) PA probes.

The environment of the experimental data acquisition is shown in Figure 11. In the service depots, the wheel runs in the designated area, and the robotic arm will carry a phased array probe to collect the testing information. The collected signals will be stored in the industrial computer for preliminary analysis like different gains to give a short judgement.

**Figure 12:**
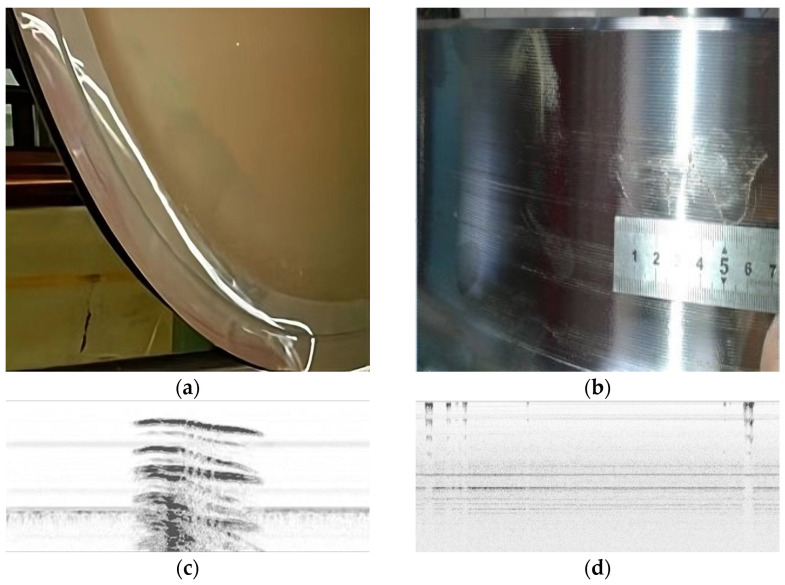
Typical defects: (**a**) wheel rim crack; (**b**) scratch; (**c**) ultrasonic b-scan defect image of rim crack; (**d**) surface and near-surface b-scan defect images.

To illustrate the practicability of the proposed method, this work briefly displays the typical real defects, including wheel rim cracks and some surface defects like scratches, with their B-scan images given below in Figure 12.

Another thing that should be taken into consideration is the variation in the depths of the scan with the different ratios of width to height. It was necessary to perform a data-preprocessing procedure. First, the images were cropped and reorganized into rectangles with an aspect ratio of 1 to the greatest extent possible, as in the algorithm for data preprocessing shown below (Algorithm 1).
**Algorithm 1:** Data Preprocessing**Input:** Original Images. **Output:** Processed Image after Data Preprocessing.1:// Obtain the width (w) and height (h) of the image2:w, h = getImageDimensions(originalImage)3:// Check if the width is greater than 3 times the height4:**if** w > 3 * h:5:// Crop the image into two halves and stack them vertically6:processedImage = cropAndStackImage(originalImage)7:**else:**8:// If width is not greater than 3 times height, no processing is done9:processedImage = originalImage10:**end if**11:// Return the processed image12:**return** processedImage

Then, the processed images needed to be labeled with different classes. Finally, the images were divided into three sets: a training set, a validation set, and a testing set with a ratio of 7:2:1, containing 10,395, 2970, and 1485 images, respectively.

### 4.2. Evaluated Index

In this work, two sets of metrics from academia and industry, respectively, are used to evaluate the model. Generally, the academic indexes that should be used are average precision (AP), recall, mAP, and frames per second (FPS); the industrial indexes always focus on the true alarm rate (TAR) and the false alarm rate (FAR) of detection.

#### 4.2.1. Academic Indexes

A confusion matrix is often used to summarize the prediction effect of classification models, as shown in Table 1.

The calculations are shown in Equations (9)–(13):

(9) $$precision=\frac{TP}{TP+FP}$$

(10) $$Recall=\frac{TP}{TP+FN}$$

(11) $$AP=\int_{0}^{1}p(r)dr$$

(12) $$mAP=\frac{1}{n}{\sum_{k=1}^{n}AP}_{k}$$

(13) $$FPS=\frac{frameNum}{elapsedTime}=\frac{N}{T}$$

#### 4.2.2. Industrial Indicators

**Figure 13:**
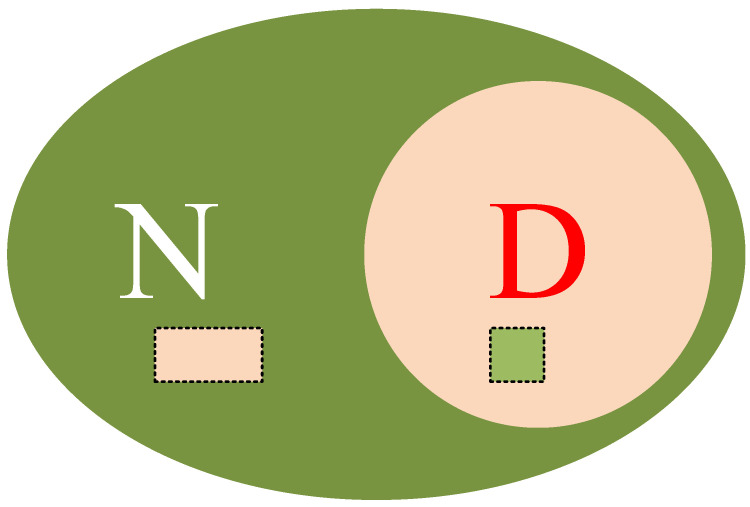
Example explaining the industrial indicators.

An example describing the true alarm rate (TAR) and false alarm rate (FAR) is provided here. Assuming there are T samples in total, and D is the number of defect samples, the rest of the samples, called N, are normal. Using an algorithm of detection, in the problem section, D − 1 out of D samples were found to be defects, and in the normal section, 2 out of N were identified as defects; an explanatory diagram is shown in Figure 13, and the calculation formulae should be like those in Equations (14) and (15).

(14) $$TAR=\frac{D-1}{D}$$

(15) $$FAR=\frac{2}{N}$$

In this example, there is one more definition to provide, that is, the miss alarm rate (MAR), which in this case is 1 out of D, as represented in Equation (16).

(16) $$MAR=1-\frac{D-1}{D}=\frac{1}{D}$$

In practice, there is a big gap between academia and industry. The requirements for statistical results are often stringent in the industrial setting, making the utilization of industrial indexes more challenging. In this work, academic metrics were adopted primarily to validate the feasibility of algorithmic improvements, serving as an assessment tool for evaluating the effectiveness of the algorithm.

### 4.3. Implementation Details

All the experiments were carried out on a computer running Windows 10. Two Nvidia GeForce RTX3090 GPUs were used, and the CPU version was Intel Core i9-1098XE. The deep learning framework was Pytorch 1.7.1, and the python version was 3.7.11. The image input was resized to 640 × 640, the batch size was 128, the optimizer was SGD, the initial learning rate was set to 0.001, the total number of iterations was set as 500 epochs, and the patience was set to 50, signifying that if there are 100 consecutive rounds without improvement, the system defaults to convergence.

### 4.4. Experiment Results

**Figure 14:**
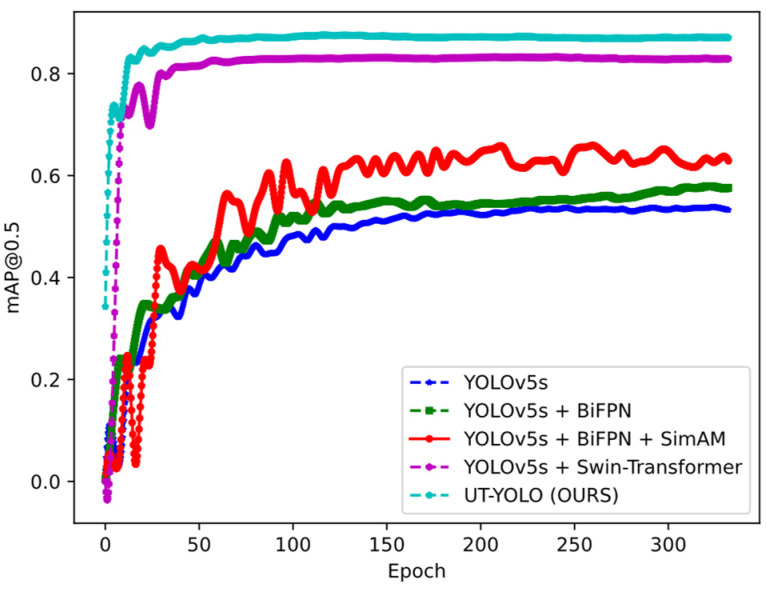
mAP@0.5 for the ablation experiment.

**Figure 15:**
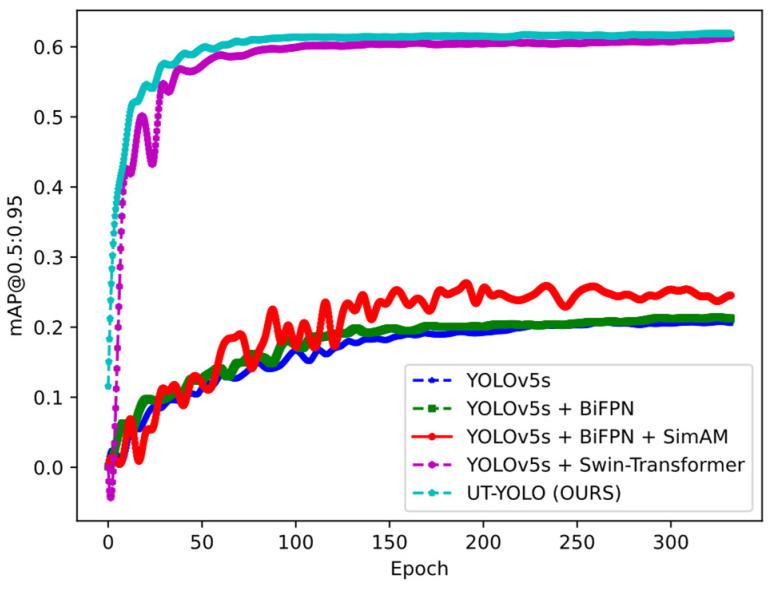
mAP@0.5:0.95 for the ablation experiment.

To evaluate the performance of the algorithm in question in terms of accuracy and speed in terms of both academic and industrial applications, UT-YOLO was compared with different algorithms. Table 2 presents the best recall, mAP@0.5, and mAP@0.5:0.95 results for each algorithm, derived from the experimental observations of the proposed model with respect to the test set. Figure 14 and Figure 15 depict the mAP@0.5 and mAP@0.5:0.95.

**Figure 16:**
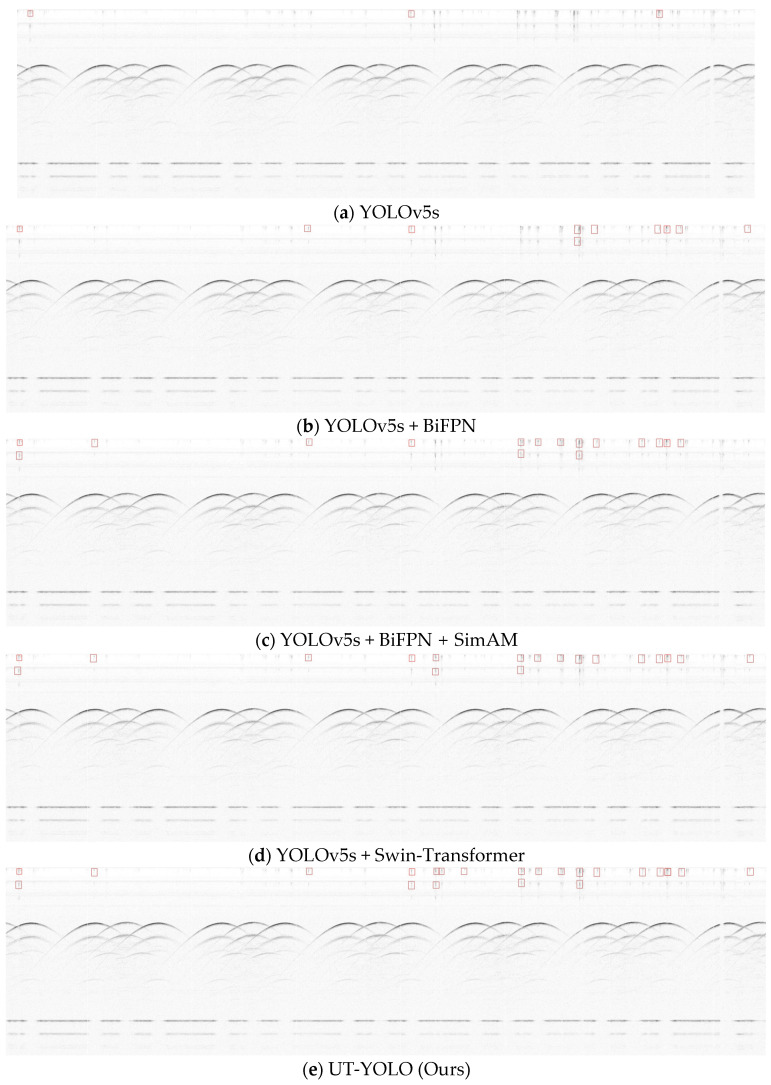
Comparison of detection results achieved using different models.

The results depicted in Figure 14 and Figure 15 show the effectiveness of UT-YOLO. Compared with the original baseline, the IoU0.5 indicator and the 0.5:0.95 indicator have been greatly improved, which is a very exciting result. In Figure 16, a comparative analysis of the detection results across different models is presented, highlighting the performance variations for a common object. Notably, the examination distinctly underscores the superior efficacy of the UT-YOLO model.

**Figure 17:**
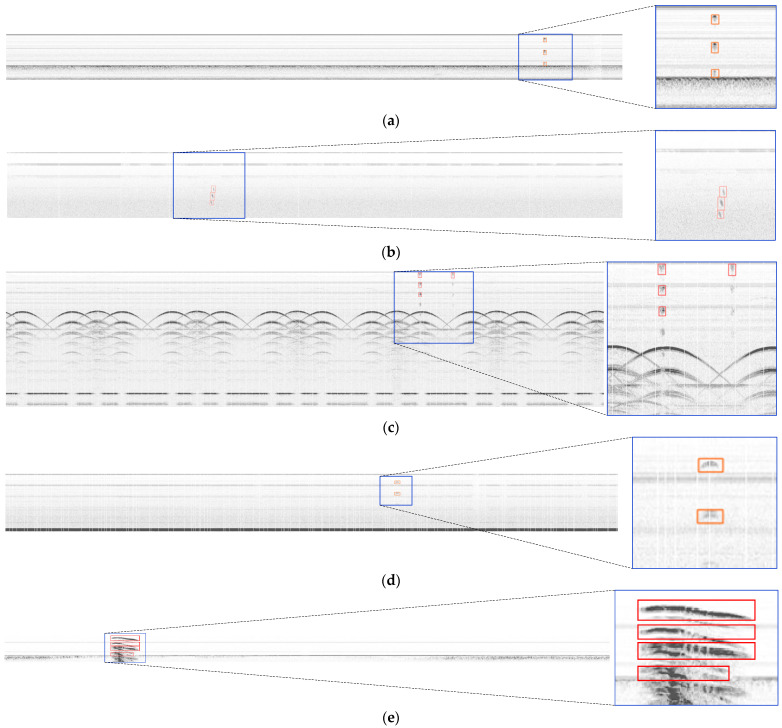
Detection results and magnified details. (**a**) surface and near-surface crack; (**b**) inner defect; (**c**) the defect near the web plate hole region; (**d**) Surface and near-surface hole-like defects; (**e**) wheel rim crack.

It can be observed in Figure 17 that the UT-YOLO model demonstrated precise localization of the defects, especially the small-sized defects. Furthermore, for defects near the wheel plate hole, the model exhibited commendable feature extraction capabilities, as evident in the outcomes presented in Figure 17. Despite the presence of some minor defects associated with background blur, the model displays robustness and can effectively detect defects, even in challenging conditions like strong background noise and electrical interference. The left side of each image is the original detection result, and the blue box on the right corresponds to the enlarged local features.

For real-world industrial inspection, additional sets of 50 defective samples and 50 non-defective samples were carefully chosen to assess the detection rate and false alarm rate. Among the 50 defective samples, consisting of a total of 6000 images, 210 were identified as defects by the UT inspectors. Using UT-YOLO, a total of 206 defect instances were successfully detected, resulting in a TAR of 98.10% (206 out of 210). Notably, the four undetected defects existed in three distinct wheelsets. In practical applications, the achieved TAR was 94% (47 out of 50), underscoring the heightened challenges faced in real-world scenarios.

Furthermore, a thorough examination of 6000 defect-free images from 50 wheelsets revealed 83 instances of false alarms, originating from 6 different wheelsets. According to statistical analysis based on the images, the calculated FAR was 1.38% (83 out of 6000). However, in actual application scenarios, the observed FAR was 12% (6 out of 50). These results emphasize the complexity and intricacies encountered in practical industrial applications, where achieving optimal detection indicators proves to be a more demanding task.

Last but not least, the efficiency of this method aligns with the demands of real-time collection and detection. In typical scenarios, a wheel completes one full rotation in about one minute. With a phased array probe utilizing 120 channels for data collection, the algorithm achieves a detection speed of approximately 2 s per wheel. Considering factors such as software response and data loading time, on-site application can accomplish the detection of a wheel and provide defect location information within 10 s, significantly diminishing the expenses associated with manual inspection.

## 5. Discussion

In this study, the application of the UT-YOLO method to ultrasonic inspection data of railway wheels was inspected, with a focus on detecting various types of defects. The evaluation of results underscores the superiority of this method, emphasizing its reliability both in field applications and on site. Among the models employed for object detection, the proposed UT-YOLO model exhibits significant advancements compared to several benchmark models. The outcomes indicate that UT-YOLO achieved a best recall of 0.94, an mAP@0.5 of 0.89, and an mAP@0.5:0.9 of 0.64, surpassing baseline YOLOv5s by 37%, 36%, and 42%, respectively. Particularly noteworthy is UT-YOLO’s substantial superiority over other comparative algorithms in terms of mAP@0.5:0.9. Moreover, UT-YOLO demonstrated the fastest speed among the evaluated models. This is attributed to its unique features, such as an added small object detection layer, an attention mechanism module, data enhancement, and data preprocessing, specially designed for ultrasonic wheel b-scan image defect detection.

While the current study has effectively addressed challenges in ultrasonic wheel detection, especially in regard to speed and accuracy, achieving promising outcomes in terms of efficiency and practical applicability, it is crucial to acknowledge the diverse nature of real-world defects. Despite this success, certain limitations may arise in addressing specific prevalent issues. In particular, some false alarms caused by external factors such as electrical interference are still relatively serious, so the data collection process is very dependent on the stability of the equipment because the surface conditions of the wheels are not the same, which will cause the data to be easily invalidated, and a large amount of noise, especially electrical noise, is generated at the same time. However, to avoid excessive investment, more targeted distinctions will be made on the algorithm side to eliminate some special interferences. This will also be reflected in future work. Therefore, future endeavors will prioritize an in-depth exploration of actual wheel inspection equipment and the quality of inspection data. These tasks will involve a meticulous classification of various defects, aiming to enhance this methodology’s performance across a broader spectrum of real-life scenarios.

## 6. Conclusions

The study presents a noteworthy breakthrough in defect detection methods utilizing ultrasound B-scan images obtained through the application of UT-YOLO. The UT-YOLO model incorporates specific modifications that significantly enhance the accuracy of defect detection, particularly in the context of B-scan images of ultrasonic defects. The notable enhancements include the integration of small-object detection layers, residual structures for feature fusion, attention mechanisms, and specialized processing methods. Collectively, these adjustments culminate in a robust model poised to address the challenges inherent in wheel defect detection in real-world ultrasonic B-scan images. When compared with the baseline YOLOv5s model, UT-YOLO demonstrated marked performance improvements. In the wheel defect dataset test results, it achieves noteworthy enhancements of 37%, 36%, and 43% in best recall, mAP@0.5, and mAP@0.5:0.95, respectively. Simultaneously, the speed of UT-YOLO reached an impressive 69FPS, indicating its capacity to operate effectively in real-time applications and making it well suited for deployment in practical scenarios.

Despite the UT-YOLO algorithm’s notable successes in UT defect detection, there are still some areas that may be further investigated and refined. To further lower the false alarm rates and missed alarm rates, future work will concentrate on distinguishing the noise and the noise-like defects as well as the whole automatic process in train wheel defect detection to achieve truly intelligent detection.

## Figures and Tables

**Table 1:** Confusion matrix.

	Actual Positive	Actual Negative
Predicted Positive	TP	FP
Predicted Negative	FN	TN

**Table 2:** Results regarding detection for the UT-B scan dataset.

Model	Best Recall	mAp@0.5	mAp@0.5:0.95	FPS
YOLOv5s	0.57	0.53	0.21	55
YOLOv5s + BiFPN	0.63	0.58	0.21	58
YOLOv5s + BiFPN + SimAM	0.67	0.63	0.25	57
YOLOv5s + Swin-Transformer	0.85	0.82	0.61	64
UT-YOLO (Ours)	**0.94**	**0.89**	**0.64**	**69**

## Data Availability

Data are contained within the article.
